# A *Rickettsiella* Bacterium from the Hard Tick, *Ixodes woodi*: Molecular Taxonomy Combining Multilocus Sequence Typing (MLST) with Significance Testing

**DOI:** 10.1371/journal.pone.0038062

**Published:** 2012-05-31

**Authors:** Andreas Leclerque, Regina G. Kleespies

**Affiliations:** Institute for Biological Control, Julius Kühn Institute (JKI) - Federal Research Centre for Cultivated Plants, Darmstadt, Germany; University of Minnesota, United States of America

## Abstract

Hard ticks (Acari: Ixodidae) are known to harbour intracellular bacteria from several phylogenetic groups that can develop both mutualistic and pathogenic relationships to the host. This is of particular importance for public health as tick derived bacteria can potentially be transmitted to mammals, including humans, where e.g. *Rickettsia* or *Coxiella* act as severe pathogens. Exact molecular taxonomic identification of tick associated prokaryotes is a necessary prerequisite of the investigation of their relationship to both the tick and possible vertebrate hosts. Previously, an intracellular bacterium had been isolated from a monosexual, parthenogenetically reproducing laboratory colony of females of the hard tick, *Ixodes woodi* Bishopp, and had preliminarily been characterized as a “*Rickettsiella*-related bacterium”. In the present molecular taxonomic study that is based on phylogenetic reconstruction from both 16 S ribosomal RNA and protein-encoding marker sequences complemented with likelihood-based significance testing, the bacterium from *I. woodi* has been identified as a strain of the taxonomic species *Rickettsiella grylli*. It is the first time that a multilocus sequence typing (MLST) approach based on a four genes comprising MLST scheme has been implemented in order to classify a *Rickettsiella*-like bacterium to this species. The study demonstrated that MLST holds potential for a better resolution of phylogenetic relationships within the genus *Rickettsiella*, but requires sequence determination from further *Rickettsiella*-like bacteria in order to complete the current still fragmentary picture of *Rickettsiella* systematics.

## Introduction

Ticks are prominently known to harbour intracellular bacteria from different taxonomic groups as, for instance, the alpha and gamma subdivisions of the proteobacteria. Whereas some of these bacteria as e.g. those of the genera *Rickettsia* or *Coxiella* are important tick-transmitted human or animal pathogens, others as e.g. those assigned to the genus *Wolbachia* are commonly referred to as endosymbionts. As a rule, the latter are transmitted transovarially and have established a putatively mutualistic interaction with the host. However, the nature of such arthropod host – bacterial endosymbiont relationships is complex, with endosymbionts being able to induce reproductive alterations in or become pathogenic for the host [1], [2].

An intracellular bacterium has previously been observed in an originally bisexual laboratory colony of the hard tick, *Ixodes woodi* Bishopp (Acari: Ixodidae), after its development into a colony of parthenogenetically reproducing females. The bacterium has been analyzed by electron-microscopy and phylogenetically by sequencing of its 16 S ribosomal RNA gene that was found most closely related to the, at the time, only available ortholog from the genus *Rickettsiella*, more exactly from the cricket pathogen *R. grylli*, and – more distantly - to orthologous sequences present in *Coxiella* genomes. In the light of these results, the *I. woodi* associated new specimen was consistently classified as a “*Rickettsiella*-related bacterium” [3].

The genus *Rickettsiella* (Philip) comprises intracellular bacteria associated with a wide range of arthropods that typically multiply in vacuolar structures within host tissue, e.g. fat body, cells and are frequently associated with protein crystals. *Rickettsiella* bacteria are mostly perceived as arthropod pathogens, but at least one case of a well documented mutualistic interaction of a *Rickettsiella* bacterium with its host - the pea aphid, *Acyrthosiphon pisum*, - has been reported [4]. The slowly proceeding *Rickettsiella* infections are cyto- and histologically complex, including polymorphic bacterial development and replication within and subsequent release from host cells, and thereby bear resemblance with rickettsial and chlamydial infections [5].

The current taxonomy of these bacteria [6] is based on a pathotype designation that is partially superposed by the morpho- and serologically founded distinction of four recognized species, namely the nomenclatural type species *Rickettsiella popilliae* (Dutky and Gooden) as well as *Rickettsiella grylli* (Vago and Martoja), *Rickettsiella chironomi* (Weiser), and *Rickettsiella stethorae* (Hall and Badgley). Several, but not all, *Rickettsiella* pathotypes have been placed in synonymy with one of the recognized species. For instance, the pathotype ‘*Rickettsiella melolonthae*’, i.e. the causative agent of the “Lorsch disease” of white grubs of the European cockchafer, *Melolontha* spp. (Coleoptera: Scarabaeidae) [7] is considered a subjective synonym of *R. popilliae* [6].

Due to their early perception as “rickettsiae of insects“, *Rickettsiella* bacteria had originally been assigned to the order *Rickettsiales* that currently belongs to the *Alphaproteobacteria* [8]. However, based on 16 S rRNA sequencing results from a strain of *Rickettsiella grylli* [9], the genus *Rickettsiella* has been re-assigned to the gamma-proteobacterial order *Legionellales* [6]. On a genomic basis, this reorganization has been confirmed [10] for a different strain of *R. grylli* isolated from the common pill-bug, *Armadillidium vulgare*, and receives additional support from the determination of 16 S rRNA-encoding sequences from further *Rickettsiella* pathotypes [11]–[15]. This group comprises several synonyms of the type species, *R. popilliae*, whereas to date no genetic data are publicly available from the species *R. chironomi* and *R. stethorae*. Moreover, there are currently four reports on *Rickettsiella*-like bacteria from different species of *Ixodes* ticks, namely from *I. woodi* [3], *I. tasmani* [16], and *I. ricinus* [17], [18]. As *Rickettsiella* bacteria, and especially pathotypes of the species *R. popilliae*, are under study as possible insect biocontrol agents, the possibility of their transmission to vertebrates, e.g. by acarid vectors, is a major risk assessment concern.

In view of the inapplicability of the classical species concept to asexually reproducing micro-organisms, comparison of orthologous gene sequences has been widely used to infer phylogenetic relationships among bacteria, and for good reason 16 S ribosomal RNA-encoding sequences have become the standard molecular chronometer in prokaryote phylogenetics [19]. However, complementary phylogenetic information derived from the analysis of additional, protein-encoding marker gene sequences, termed multilocus sequence typing (MLST) [20], is often useful or required to improve resolution at lower taxonomic ranks, e.g. where species or subspecies delineation is intended, or to evaluate and eliminate phylogenetic inconsistencies due to, for instance, lateral gene transfer. Recently, a comparative genomics approach has identified MLST schemes for numerous bacterial genera and species, but without considering members of the taxonomic order *Legionellales* [21].

Within the taxonomic family *Coxiellaceae* that currently comprises both the genera *Coxiella* and *Rickettsiella*, several protein-encoding genes have been investigated as possible markers for phylogenetic studies beyond the 16 S rRNA gene level [12], [17], [22], [23], but often with only limited success. For the genus *Rickettsiella*, a systematic evaluation of possible phylogenetic markers that operate reasonably well at the infra-generic level has revealed a set of four MLST markers comprising the *ftsY* gene encoding the bacterial homolog of subunit alpha of the eukaryotic signal recognition particle receptor involved in protein translocation, the *gidA* gene encoding glucose inhibited cell division protein A, the *rpsA* gene encoding the 30 S ribosomal protein S1, and the *sucB* gene encoding dihydrolipoamide succinyltransferase [24]. The *ftsY* gene had been identified previously as the most appropriate single gene marker for the estimation of the G+C content of prokaryotic genomes [25].

Ideally, phylogenetic reconstruction including confidence limit assessment for tree substructures, e.g. by bootstrapping analysis, provides information on the best phylogenetic tree(s) identified, i.e. on the most likely or most parsimonious representation(s) of the sequence data underlying the reconstruction. In contrast, it does not provide any immediate information on how much worse the respective second-best trees are. It is, basically, this type of additional information that can be assessed using likelihood-based significance testing employing an algorithm that with respect to a set of sequence data i) attributes a likelihood value to each single tree topology from a set of candidate trees, ii) ranks them by decreasing likelihood, and iii) rejects those tree topologies that - with respect to an exogenously established significance threshold - are significantly worse interpretations of the underlying sequence data than the most likely tree [26]. Following this rationale, a reconstructed phylogeny is considered the more reliable the more clearly alternative second-best topologies are rejected.

In the study presented here, the phylogeny and molecular taxonomy of the previously analyzed “*Rickettsiella*-related bacterium” associated with a parthenogenetically reproducing monosexual colony of the hard tick, *Ixodes woodi*, was re-examined in greater depth, with particular respect to the availability of additional sequence data from *Rickettsiella*-like bacteria as well as a more advanced infra-generic classification methodology that combines 16 S rRNA based phylogenetic reconstruction to both MLST and likelihood-based significance testing.

## Methods

### DNA Preparation

Genomic DNA was prepared from individual adult female ticks. Animals were immobilized in dental wax (Surgident, Miles, South Bend, IN, USA) and tissues extirpated and washed in Hank's balanced saline solution. DNA was extracted from the extirpated tissues using the Puregene DNA isolation kit (Gentra Systems, Minneapolis, MN, USA) according to the solid tissue protocol provided by the manufacturer.

### PCR Amplification

PCR amplifications of both the 16 S rRNA gene and the MLST markers were performed in duplicate from two DNA samples, “Iw1” and “Iw3”, corresponding to two different *I. woodi* individuals from the same colony, with Phusion High-Fidelity DNA polymerase (New England Biolabs) using the oligonucleotide primers and annealing temperatures described in Table 1 in a reaction comprising an initial denaturation step of 2 min at 94°C preceding 30 reaction cycles of a 15 sec denaturation step at 94°C, a 30 sec annealing step at the primer pair specific temperature and a 2 min elongation step at 72°C, followed by a final elongation step of 5 min at 72°C. For each marker gene, PCR products from both independent amplification reactions were purified by passage over a Qiaquick column (Qiagen) and sequenced on both strands (SeqLab, Göttingen, Germany). Raw sequence data were analyzed including base call quality control, combined into a single consensus sequence and translated into peptide sequences using the DNA Strider 1.3 software tool.

**Table 1:** MLST marker genes.

Marker gene	Gene product	PCR primers used for amplification, upper line: forward primer, lower line: reverse primer	T_A_	Length of amplified sequence	GenBank accession number
*rrs*	16 S ribosomal RNA	5′- TGAAGAGTTTGATCCTGGCTCAG 5′-CCTACGGCTACCTTGTTACGACTT	52°C	1,538 bp	AF383621
*ftsY*	signal recognition particle- receptor, subunit alpha	5′-AGYTTNCKNCCNCKCCAYTGNCCNCC 5′-AGRTCRAANCCNCCNCCRAACCACC	50°C	996 bp	JQ740154
*gidA*	glucose inhibited cell division protein A	5′-GAATACAATGGCGTACATTGAATGC 5′-AAGACGGAAAAAGATCGCGTGGATC	50°C	786 bp	JQ070345
*rpsA*	30 S ribosomal protein S1	5′-AAAGTAAAAGGCGGTTTTACNGTNGA 5′-GAAATACGTTCTCGTTCNGGRTCDAT	48°C	879 bp	JQ070346
*sucB*	Dihydrolipoamide succinyl- transferase component E2	5′-TAGAAGTACCGGCAYCCGCYGACGG 5′-ACATCATCGGTCGAATAACCACTTG	52°C	924 bp	JQ070347

“T_A_” designates the annealing temperature used for amplification with the respective primer pair. 16 S rRNA gene primers are from Weisburg et al. [49], MLST marker primers from Leclerque et al. [24].

### Sequence Data Analysis

Orthologous GenBank database entries were identified using the BlastN or tBlastN software tools [27], [28] for ribosomal RNA and protein-encoding marker sequences, respectively. For the reconstruction of a 16 S rRNA phylogeny BlastN hits were ranked according to their maximal sequence identity, i.e. independent of the respective percentage sequence coverage, with a value of <90% being applied as a cut-off criterion. For groups of sequences stemming from the same source, the single sequence entry displaying highest maximum identity was retained for phylogenetic analysis.

Sequence alignments were performed by means of the CLUSTAL W function [29] of the MEGA 4 program [30] using an IUB DNA or a Gonnet protein weight matrix, respectively. Protein-encoding markers were codon aligned at the deduced amino acid sequence level. The Tree-Puzzle 5.2 software [31] was used to estimate data set specific parameters as nucleotide and amino acid frequencies, the percentage of invariable sites, transition/transversion ratios and the α parameter for the Γ-distribution based correction of rate heterogeneity among sites. Pairwise sequence identity percentages were assessed from p-distance matrices calculated in MEGA 4 from unfiltered nucleotide alignments under pairwise deletion of alignment gaps and missing data.

### Phylogenetic Reconstruction

For phylogenetic reconstruction from nucleotide sequence alignments, the most appropriate models of DNA sequence evolution were chosen according to the rationale outlined by Posada and Crandall [32]. Organism phylogenies were reconstructed with the Maximum Likelihood (ML) method as implemented in the PhyML software tool [33] using the HKY model of nucleotide substitution [34]; protein-encoding nucleotide data were filtered by systematic suppression of third codon positions. For the 16 S rRNA marker, additional Neighbor Joining (NJ) and Minimum Evolution (ME) phylogenies were reconstructed in MEGA 4 from unfiltered nucleotide sequence data under, respectively, the MCL [35] and the K2P [36] models of nucleotide substitution. For protein-encoding markers, NJ and ME phylogenies were generated from hypervariability filtered nucleotide data using a modified Nei-Gojobori model [37] with Jukes-Cantor corrected rates of non-synonymous substitution. Moreover, organism phylogenies were reconstructed from amino acid sequence alignments using the JTT [38] model of substitution with the ML, NJ, and ME methods. In all cases, a Γ-distribution based model of rate heterogeneity [39] allowing for eight rate categories was assumed. Tree topology confidence limits were explored in non-parametric bootstrap analyses over 1,000 pseudo-replicates. The consistency of the thereby generated phylogenetic reconstruction results from ribosomal RNA encoding sequences was assessed by alternative alignment using the RNA alignment tool of the T-Coffee software package [40], [41]. Consensus tree topologies were generated by means of the Consense module of the Phylip 3.6 software package [42].

### Significance Testing

Likelihood-based significance testing of tree topologies was performed by the pairwise one-sided Kishino-Hasegawa (1sKH) test [31], i.e. a modification of the SH test [43], that has been shown to be superior to the original two-sided Kishino-Hasegawa (2sKH) test [44] if evaluated tree topology sets are permutatively incomplete, and in particular if so due to previous ML-based selection [26]. Candidate tree topologies for significance testing were generated manually in Newick format. The 1sKH test was performed as implemented in the Tree-Puzzle 5.2 software package applying a 5% significance threshold. For protein-encoding markers, candidate topologies were evaluated against both hypervariability filtered nucleotide and amino acid sequence alignments.

## Results

**Figure 1:**
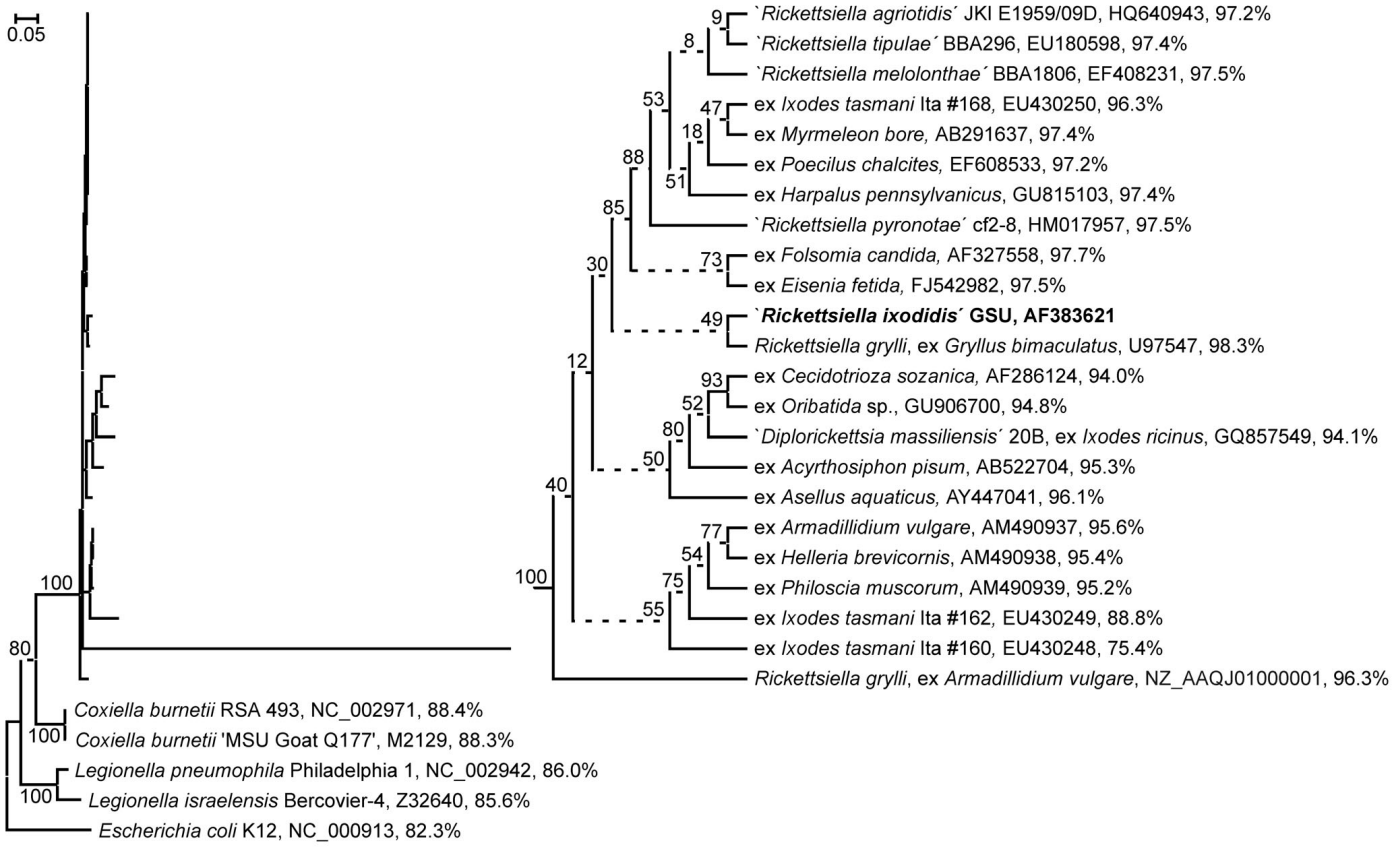
Bacterial ML phylogeny generated from 16 S ribosomal RNA encoding sequences. Terminal branches are labelled by genus and species, pathotype, strain and/or original host designations, GenBank accession numbers as well as pairwise nucleotide sequence identity percentages as calculated from a p-distance matrix with respect to the ‘*Rickettsiella ixodidis*’ GSU sequence. Numbers on internal branches indicate bootstrap support values; branches that do not receive >90% bootstrap support are represented by dashed lines. The phylogram has been rooted using *Escherichia coli* as outgroup. The size bar corresponds to 5% sequence divergence. To enhance resolution, the upper clade of the phylogram that comprises *Rickettsiella*-like bacteria, has been extended into a cladogram. GenBank accession numbers AM490937-39 and EU430248-50 designate partial 16 S rRNA gene sequences comprising only 40–70% of the complete 16 S rRNA marker sequence. The tree has been reconstructed from ClustalX aligned sequences; an essentially identical ML tree has been generated from a T-Coffee based nucleotide sequence alignment (not shown).

DNA sequencing confirmed the previously determined 16 S rRNA gene sequence from the *Rickettsiella*-related bacterium from all *I. woodi* samples investigated. When used as query sequence for a BlastN search across the GenBank nucleotide database, this sequence identified 81 further GenBank entries displaying a maximal sequence identity of at least 90%. The sequence with the highest maximal identity score (97%) stemmed from the aforementioned endosymbiont of the pea aphid. After elimination of data redundancy due to multiple entries from the same source, a set of 22 orthologous sequences was retained for phylogenetic reconstruction. Importantly, this data set contains all currently available 16 S rRNA gene sequences that i) with respect to the query sequence display a maximal sequence identity percentage superior to the respective value (86%) established for the *Coxiella burnetii* ortholog and ii) stem from an arthropod related source, i.e. are not derived from mere environmental, e.g. soil or water, samples. Moreover, for comparison across the order *Legionellales*, two *Coxiella* and two *Legionella* orthologs were included into the data set, and the *E. coli* 16 S rRNA gene was used as outgroup. Figure 1 presents the ML phylogeny reconstructed from the alignment of this sequence set.

There are two prominent features of this 16 S rRNA gene based phylogenetic tree that are essentially confirmed by the corresponding NJ and ME phylogenies (data not shown). Firstly, arthropod-associated *Rickettsiella*-like bacteria form a distinct, maximally bootstrap supported clade that unequivocally separates them from their closest neighbours, the genera *Coxiella* and *Legionella*. This clade lacks an obvious subdivision into sufficiently bootstrap-supported sub-clades and comprises 16 S rRNA genes that – with exception of two 16 S rRNA partial sequences from *Rickettsiella*-like bacteria found in *I. tasmani* - display >93% of pairwise sequence identity (data not shown). As the “*Rickettsiella*-related bacterium” from *I. woodi* is firmly located within this presumed *Rickettsiella* clade, we will henceforward refer to it, for purposes of discussion, as the *Rickettsiella* pathotype ‘*Rickettsiella ixodidis*’.

Moreover, despite extensive sequence discovery during the past decade, ‘*R. ixodidis*’ appears still most closely related to the first genetically analyzed *Rickettsiella grylli* strain obtained from the cricket, *Gryllus bimaculatus*. However, the bootstrap value supporting the corresponding branch is low (49%).

**Figure 2:**
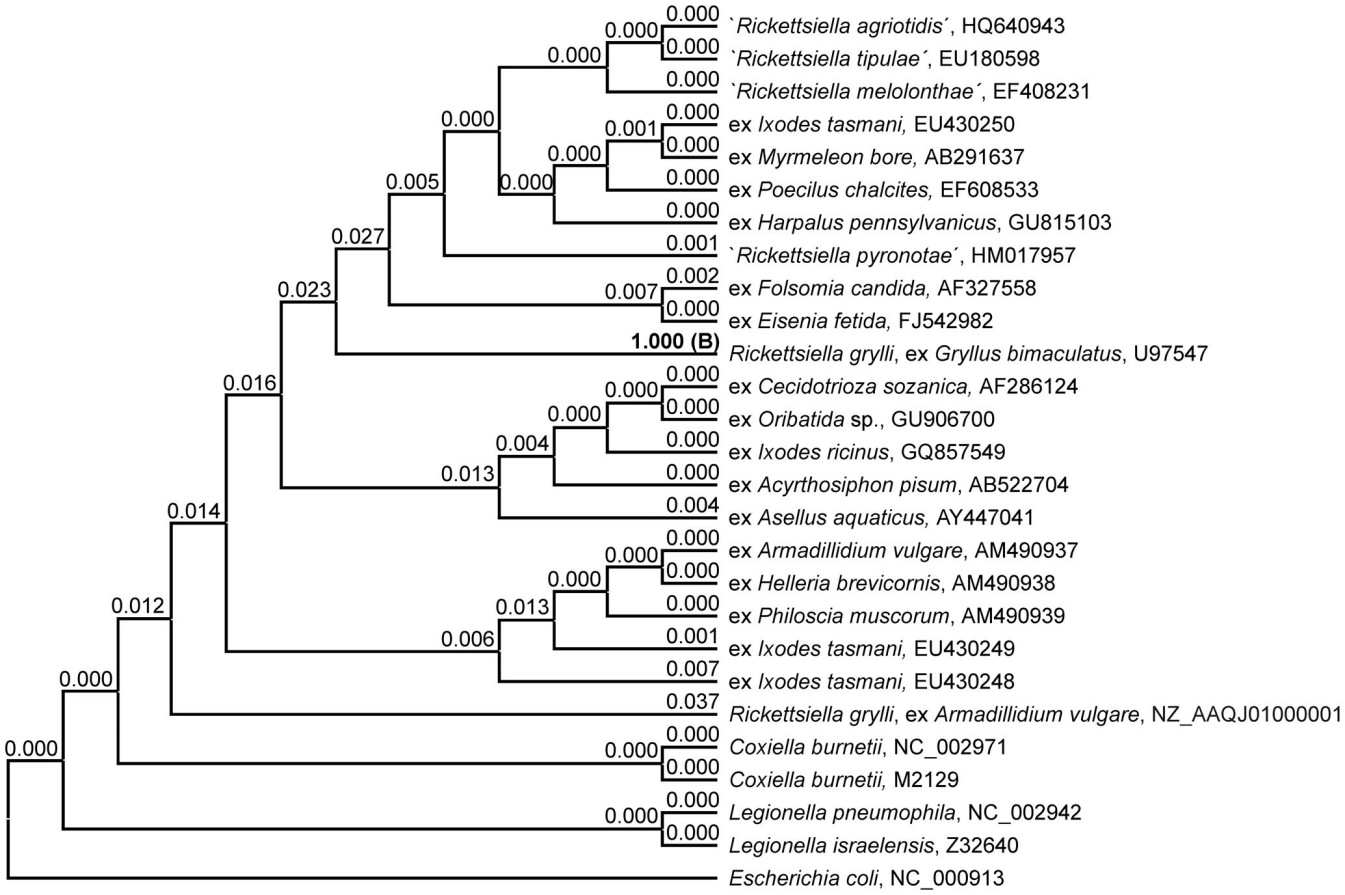
Significance testing evaluating the 16 S rRNA gene based taxonomic assignment of ‘*Rickettsiella ixodidis*’. Cladogram presenting the backbone tree topology generated by pruning off the terminal ‘*Rickettsiella ixodidis*’ branch from the 16 S rRNA gene based ML phylogeny of Figure 1. Terminal branches are labelled by genus and species, pathotype and/or original host designations as well as GenBank accession numbers. A set of 51 candidate topologies to be evaluated by the 1sKH test was generated by re-grafting the ‘*R. ixodidis*’ branch to any of the 51 branches of the backbone topology. Numbers on branches indicate the p-value attributed by the 1sKH test with respect to the 16 S rRNA gene alignment to the candidate topology that carries ‘*R. ixodidis*’ grafted to the respective branch. Candidate topologies that remain unrejected under application of a 5% significance threshold are indicated by the p-value printed in bold type; “B” designates the most likely tree.

Significance testing designed to evaluate if the location of ‘*R. ixodidis*’ in the 16 S rRNA ML phylogeny corresponds to a significantly better description of sequence data than any of the possible further 50 locations generated a highly differential outcome (Figure 2). Expectedly, the ML phylogeny shown in Figure 1 was identified as best tree, while all further candidate topologies were rejected by the 1sKH test. The second-best tree receiving the highest score (3.7%), i.e. candidate topology #42 that relates ‘*R. ixodidis*’ to the second available *R. grylli* sequence, clearly failed to pass the 5% significance threshold.

In order to obtain 16 S rRNA independent information on the infra-generic taxonomic position of ‘*R. ixodidis*’, the complete *ftsY* gene coding sequence as well as internal partial sequences of the marker genes *gidA*, *rpsA*, and *sucB* (Table 1) were amplified and determined from DNA samples extracted from infected tissues of *I. woodi*. In all cases, corresponding sequences obtained from samples “Iw1” and “Iw3” were identical. When used as query in a tBlastN search for homologous GenBank entries, each of the four amplified MLST marker sequences identified as best hits the corresponding orthologs from the *R. popilliae*-synonymized pathotypes ‘*R. melolonthae*’ and ‘*R. tipulae*’; for the *ftsY* marker, two additional orthologs from the pathotypes ‘*R. pyronotae*’ and ‘*R. agriotidis*’ were found to display comparable sequence similarity. Expectedly, from a separate search across whole genome sequencing projects submitted to the GenBank database, the four MLST marker sequences from ‘*R. ixodidis*’ identified their respective orthologs from the genome of a strain assigned to the species *R. grylli*, but originally obtained from the pill bug, *Armadillidium vulgare*. Further sequences attributable to *Rickettsiella*-like bacteria were not identified; in particular, the algorithms failed to identify a previously published *ftsY* gene partial sequence from a *Rickettsiella*-like endosymbiont of *Ixodes ricinus* ticks that had been described as representative of a new genus, termed ‘*Diplorickettsia*’ [17].

Estimation of the genomic G+C content from the amplified ‘*R. ixodidis*’ *ftsY* gene according to the rationale developed by Fournier et al. [25] generates a calculated G+C value (38.2%) well in line with both the real G+C content of 37% determined for the *R. grylli* genome project strain and the G+C content estimates (36%–41%) obtained from DNA hybridization studies with *Rickettsiella* bacteria [45].

**Figure 3:**
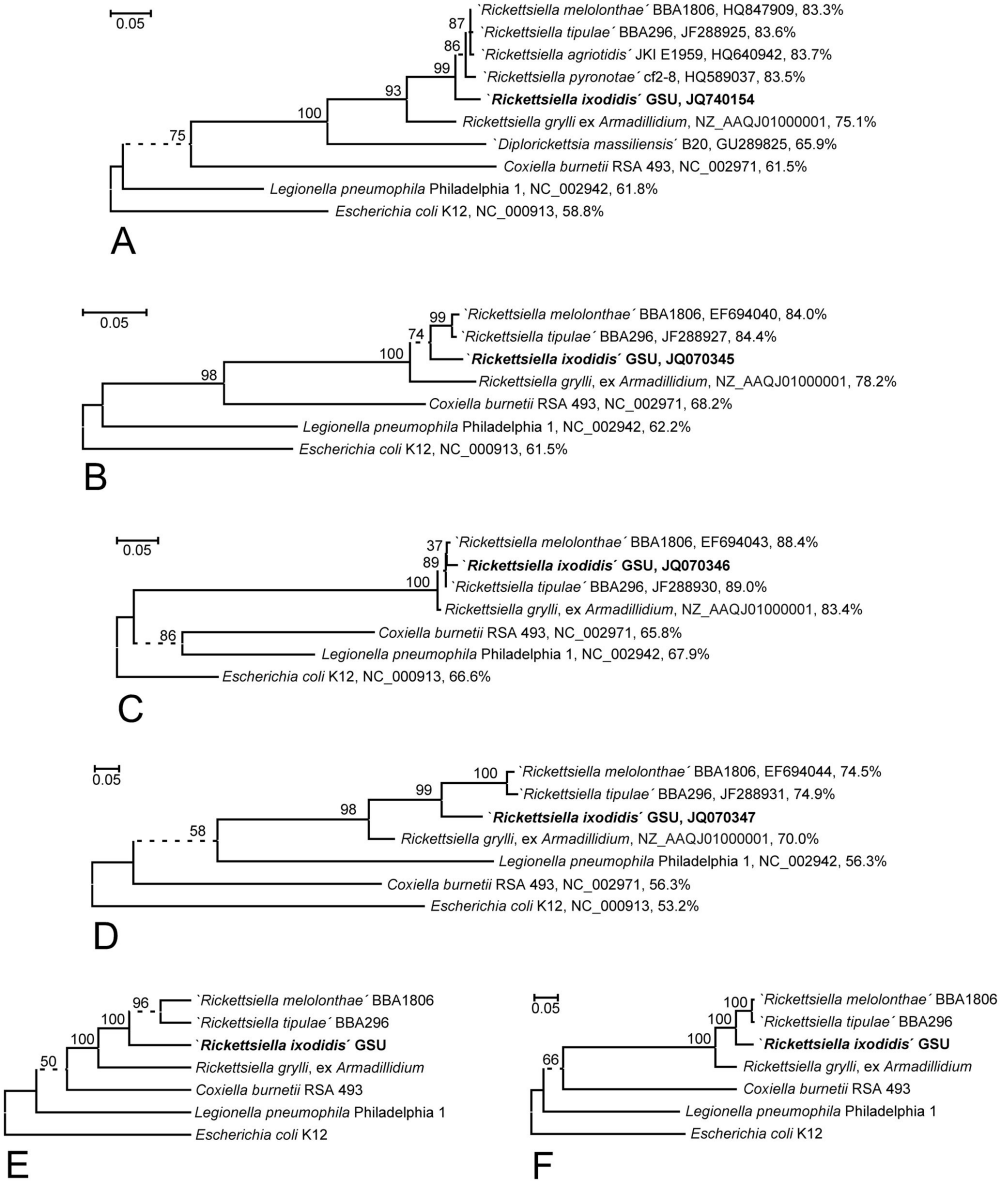
Bacterial phylogenies generated from MLST marker sequence alignments. **A–D, F:** Bacterial ML phylogenies generated from hypervariability filtered *ftsY* (**A**), *gidA* (**B**), *rpsA* (**C**), and *sucB* (**D**) gene sequences or a concatenation of these (**F**). Terminal branches are labelled by genus and species, pathotype and/or strain designations, GenBank accession numbers, and pairwise nucleotide sequence identity percentages respect to the ‘*Rickettsiella ixodidis*’ GSU sequence as calculated from a p-distance matrix with. Numbers on internal branches indicate bootstrap support values; branches that do not receive >90% bootstrap support are represented by dashed lines. Trees were rooted using *Escherichia coli* as outgroup. The size bar corresponds to 5% sequence divergence. **E:** Extended majority rule consensus tree topology generated from 24 phylogenies reconstructed by the ML, ME, or NJ method from hypervariability filtered nucleotide or deduced amino acid sequence alignments of the *ftsY*, *gidA*, *rpsA*, or *sucB* marker. For compatibility with the other markers, specimens ‘*R. agriotidis*’, ‘*R. pyronotae*’ and ‘*Diplorickettsia massiliensis*’ were removed from the *ftsY* data sets prior to single phylogeny reconstruction. Terminal branches are labelled by genus and species, pathotype and/or strain designations. Internal branches collapsing under the strict consensus criterion are represented by dashed lines; the frequency of occurrence across the aggregated set of phylogenies is indicated as a percentage value on top of the respective branch.

When the MLST marker sequences from ‘*R. ixodidis*’ were compared to the identified orthologs as well as representatives of the most closely related genera *Coxiella* and *Legionella*, phylogenetic reconstruction gave rise to the trees presented in Figure 3. The four single MLST marker ML phylogenies (Figure 3A–D) coincide with the respective NJ and ME phylogenies (data not shown) in placing all *Rickettsiella-*like bacteria in one clade that receives near maximal (98–100%) bootstrap support thereby delineating the genus *Rickettsiella* (as well as, for the *ftsY* marker, the proposed genus ‘*Diplorickettsia*’) unequivocally from both *Coxiella* and *Legionella*. Within the *Rickettsiella* clade, *R. grylli* is in all trees consistently located in an outgroup position with respect to a sub-clade comprising ‘*R. melolonthae*’, ‘*R. tipulae*’, and ‘*R. ixodidis*’. The topology of this sub-clade varies among the single marker trees, but both the consensus tree combining the information from the MLST marker sequence alignments (Figure 3E) and the ML tree generated from concatenated marker sequence data (Figure 3F) reveal a strong overall tendency towards a tight clustering of ‘*R. melolonthae*’ and ‘*R. tipulae*’ and their much looser association with ‘*R. ixodidis*’, as implemented with maximal bootstrap support in the *sucB* ML phylogeny (Figure 3D).

**Figure 4:**
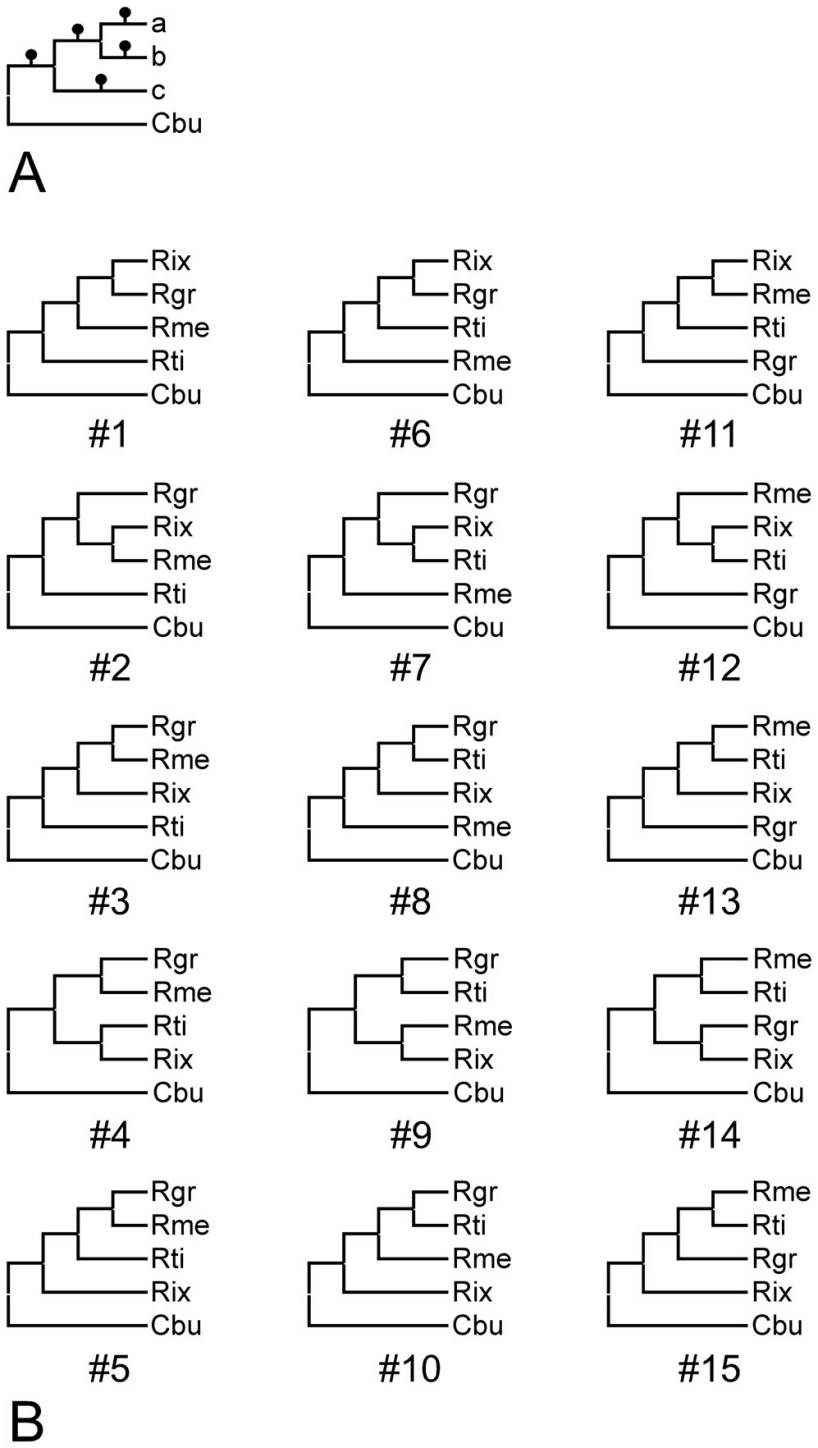
Generation of candidate tree topologies for likelihood-based significance testing based on MLST markers. 15 candidate topologies were generated from a four organism basic tree (**A**) comprising *Coxiella burnetii* (acronym “Cbu”) as outgroup together with three *Rickettsiella* strains in positions a–c. The candidate topologies tested (**B**) were generated from this basic tree by the following steps: 1. Permutative addition of three *Rickettsiella* strains representing the species *Rickettsiella grylli* (“Rgr”) and the pathotypes ‘*Rickettsiella melolonthae*’ (“Rme”) and ‘*Rickettsiella tipulae*’ (“Rti”) to positions a–c generates a maximum of six tree topologies that are pair-wise identical, i.e. a total of three different topologies. 2. Subsequent addition of the pathotype ‘*Rickettsiella ixodidis*’ (“Rix”) to any of the five branches indicated by a black dot in any of the previously generated trees.

Phylogenetic reconstruction was complemented with significance testing for the MLST markers in a model system (Figure 4A) comprising ‘*R. ixodidis*’ together with the three *Rickettsiella* specimens identified by all markers in the GenBank database and *Coxiella burnetii* as outgroup. The 15 candidate tree topologies shown in Figure 4B were evaluated against the hypervariability filtered nucleotide and amino acid sequence data from the *ftsY*, *gidA*, *rpsA*, and *sucB* markers as well as the (unfiltered) 16 S rRNA encoding nucleotide sequence data. The 1sKH test results (Table 2) indicate that in line with expectations from previous analysis [24], *gidA* and *sucB* are more phylogeny informative than the *ftsY*, *rpsA* (data not shown) and the 16 S rRNA markers that do not generate a differential 1sKH test outcome: for the *ftsY* marker only three and for the *rpsA* and 16 S rRNA markers none of the 14 second-best trees are rejected, i.e. found significantly worse interpretations of the respective sequence data than the designated marker-specific best tree. In contrast, significance test results are highly differential for both *sucB* derived data sets, with all topologies but the most likely tree (#13) being rejected. The 1sKH test outcome for the *gidA* marker is intermediately differential (Table 2). Tree #13, the only candidate tree topology found consistent with *sucB* sequence data, places ‘*R. ixodidis*’ into a sister group position with respect to the *R. popilliae*-synonyms, with the *R. grylli* strain from the pill-bug being more distantly related, as is consistent with the MLST phylogenies in Figure 3.

**Table 2:** Significance testing results for 16 S rRNA and MLST markers in a five organism model.

Candidate tree topology number	*gidA* filtered nucleotide	*gidA* deduced amino acid	*sucB* filtered nucleotide	*sucB* deduced amino acid	16 S rRNA *(R. grylli*, ex *Armadillidium)*	16 S rRNA *(R. grylli*, ex *Gryllus)*
01	0.055 (U)	0.205 (U)	0.000 (R)	0.008 (R)	0.317 (U)	0.074 (U)
02	0.054 (U)	0.104 (U)	0.000 (R)	0.000 (R)	0.056 (U)	0.001 (R)
03	0.050 (R)	0.116 (U)	0.000 (R)	0.000 (R)	0.072 (U)	0.002 (R)
04	0.042 (R)	0.057 (U)	0.000 (R)	0.000 (R)	0.052 (U)	0.001 (R)
05	0.045 (R)	0.043 (R)	0.000 (R)	0.000 (R)	0.057 (U)	0.001 (R)
06	0.031 (R)	0.057 (U)	0.000 (R)	0.011 (R)	**1.000 (B)**	0.118 (U)
07	0.031 (R)	0.055 (U)	0.000 (R)	0.000 (R)	0.056 (U)	0.000 (R)
08	0.027 (R)	0.047 (R)	0.000 (R)	0.001 (R)	0.073 (U)	0.000 (R)
09	0.019 (R)	0.048 (R)	0.000 (R)	0.000 (R)	0.061 (U)	0.000 (R)
10	0.053 (U)	0.031 (R)	0.000 (R)	0.001 (R)	0.063 (U)	0.000 (R)
11	0.032 (R)	0.083 (U)	0.000 (R)	0.002 (R)	0.060 (U)	0.001 (R)
12	0.040 (R)	0.102 (U)	0.000 (R)	0.000 (R)	0.053 (U)	0.000 (R)
13	0.325 (U)	**1.000 (B)**	**1.000 (B)**	**1.000 (B)**	0.267 (U)	0.879 (U)
14	0.244 (U)	0.134 (U)	0.045 (R)	0.050 (R)	0.202 (U)	**1.000 (B)**
15	**1.000 (B)**	0.133 (U)	0.041 (R)	0.047 (R)	0.294 (U)	0.846 (U)

1sKH test results across the 15 candidate tree topologies presented in Figure 4B with respect to different marker sequence alignments. For the protein-encoding marker genes *gidA* and *sucB* both the hypervariability filtered nucleotide and the deduced amino acid sequence levels were explored. For the 16 S rRNA marker, two data sets comprising sequences from different *Rickettsiella grylli* strains were analyzed. The p-values attributed to a tree topology by the 1sKH test are interpreted under assumption of an exogenous 5% significance threshold as “B” for a data set specific best tree, “U” for an unrejected second-best tree, and “R” for a rejected tree. Data set specific best trees are indicated in bold type.

Moreover, the same 15 candidate topologies were evaluated against an alternative set of 16 S rRNA gene sequence data, generated by replacement of the *Armadillidium* derived *R. grylli* strain by the *R. grylli* isolate from *G. bimaculatus*. The 1sKH test result for this new 16 S rRNA gene alignment (Table 2) is strikingly different in i) designating candidate topology #14 as best tree and in ii) rejecting the majority (10/14) of second-best trees. In candidate tree #14, ‘*R. ixodidis*’ tightly clusters with *R. grylli*, i.e. the strain from *G. bimaculatus*, as is consistent with the 16 S rRNA gene based 1sKH test results presented in Figure 2.

## Discussion

As part of the original description of the hard tick associated bacterium under study, its 16 S rRNA gene sequence had been determined and compared to a set of orthologous sequences from selected representatives of the main groups of bacteria commonly known to be associated with ticks. As a result, it had become clear that the new specimen clustered among the *Gammaproteobacteria* and was, therefore, different from the *Alphaproteobacteria*, e.g. the *Rickettsiales*, as well as the *Chlamydiales*. Within the *Gammaproteobacteria*, the new sequence had turned out to be most closely related to the ortholog from a *R. grylli* strain isolated from the cricket, *G. bimaculatus*. However, on the basis of the at that time available sequence data, it had seemed premature to decide about the genus-level taxonomic classification of the bacterium from *I. woodi*, and it had consistently been referred to as a “*Rickettsiella-*related bacterium” [3].

In the sequel of one decade of 16 S rRNA sequence discovery, the alternative approach chosen here consisted in the identification of all orthologous sequences of arthropod-related origin not more distantly related to the query than *Coxiella burnetii*. The phylogeny reconstructed from these sequences (Figure 1) displays a distinct clade that comprises all identified *Rickettsiella*-like bacteria and is clearly delineated from the neighbouring *Coxiella* and *Legionella* clades. The lack of a well-supported sub-clade delineation that would motivate a further taxon distinction within the clade suggests referring to the entire clade as representation of the taxonomic genus *Rickettsiella*. All pairs of full-length 16 S rRNA marker sequences comprised in the clade, including the *rrs* gene from the tick-associated bacterium that – in view of 16 S rRNA gene data somewhat surprisingly – had previously been described as first representative of the new genus ‘*Diplorickettsia*’, are at least 93% identical. In a signpost paper [46], Stackebrandt and Goebel have proposed a <97% 16 S rRNA gene sequence identity threshold for taxonomic *species* delineation, implying a considerably lower value for 16 S rRNA-based *genus* delineation. Today, different values are in practical use for different groups of bacteria, e.g. a <95% threshold for genus and <98.5% for species delineation within the *Chlamydiales* [47]; the genus *Legionella* comprises specimens that share at least 93% 16 S rRNA sequence identity [48]. As sequence diversity across the clade comprising *Rickettsiella*-like bacteria including ‘*Diplorickettsia*’ is not larger than this, it appears most parsimonious – on the basis of this genetic analysis - to refer to all organisms comprised as members of the same taxonomic genus, i.e. *Rickettsiella*, and to the bacterium from *I. woodi* as a *Rickettsiella* pathotype, ‘*Rickettsiella ixodidis*’. Moreover, the genomic G+C content estimated from the *ftsY* gene sequence of the bacterium under study is consistent with this generic classification.

Within the *Rickettsiella* clade, ‘*R. ixodidis*’ shows the highest level of pairwise sequence identitiy (98.3%) to and clusters with a *R. grylli* strain from *G. bimaculatus*. Despite the low bootstrap support for the respective branch of the ML phylogeny (Figure 1), the clustering is marked highly significant by the 1sKH test (Table 2). Consequently, it might seem a straightforward conclusion to refer to the pathotype ‘*R. ixodidis*’ as a new strain of the species *R. grylli*. However, the situation is complicated by the rather distant relationship of ‘*R. ixodidis*’ to the second available *R. grylli* strain as indicated by a 1sKH score below the rejection threshold for the corresponding candidate tree topology (#42) and a pairwise sequence identity of only 96.3%, i.e. less than between ‘*R. ixodidis*’ and *R. popilliae*-synonyms as ‘*R. melolonthae*’ and ‘*R. tipulae*’ (97.2–97.5%).

Additional protein-encoding markers were employed in order to study these relationships at higher resolution, but under the important limitation that the corresponding MLST marker sequences are at present available from only one of both *R. grylli* strains. However, results in Figure 3 and Table 2 make it sufficiently clear that the *sucB* and *gidA* sequences are phylogenetically more informative than the 16 S rRNA marker and might be the preferred genetic tool to further elucidate phylogenetic relationships within the genus *Rickettsiella*. Moreover, results of phylogenetic reconstruction and significance testing as well as the comparison of pairwise sequence distances for at least the *sucB* marker unequivocally corroborate that ‘*R. ixodidis*’ is more closely related to ‘*R. melolonthae*’ and ‘*R. tipulae*’ than to the *R. grylli* genome project strain from the pill bug.

Evaluation of 16 S rRNA sequences in the same significance test model confirms the above result that ‘*R. ixodidis*’ is most closely related to the *R. grylli* strain from *G. bimaculatus*. Consequently, both specimens should be co-assigned. Moreover, the elevated (98.3%) sequence identity value for both specimens' 16 S rRNAs is highly suggestive of their co-assignment to the same species.

Which species would that be? As both presumed *R. grylli* strains share only a rather low 16 S rRNA similarity (96.4%) and, by the same criterion, appear both more closely related to the *R. popilliae* synonyms ‘*R. melolonthae*’ and ‘*R. tipulae*’ than to each other, it is hardly possible that both equally well represent the species *R. grylli*. As for *R. grylli* no type strain as been deposited, the question cannot be solved by genetic comparison to the standard. Given this situation, the *R. grylli* strain from *G. bimaculatus* appears the more convincing representative of this species, because it has been the first genetically described *Rickettsiella* bacterium [9] and coincides in its species and pathotype assignments. In contrast, it is not clear on which basis the *Rickettsiella* genome project strain from *Armadillidium vulgare* has been assigned to the species *R. grylli*, whereas the independently existing pathotype ‘*Rickettsiella armadillidii*’ has in the past been considered a synonym of both the species *R. grylli* [8] and *R. popilliae* [6]. Consequently, we propose to refer to the *R. grylli* strain from *G. bimaculatus* as the more authentic representative of this species. According to the above analyses this strain and the *Rickettsiella* bacterium from the hard tick, *I. woodi*, should be co-assigned at the species level. We therefore conclude that the *Ixodes* derived bacterium that has preliminarily been described as the pathotype ‘*R. ixodidis*’, should be referred to as a strain of the taxonomic species *Rickettsiella grylli*.

A genetic survey of bacteria associated with *Ixodes tasmani* ticks collected from Koala (*Phascolarctos cinereus*) in Australia [16] has identified three *Rickettsiella*-like 16 S rRNA sequences. Interestingly, one of these (sample “Ita #160”) has originally been reported i) to be most closely related to the orthologs from both the *R. grylli* strain from *G. bimaculatus* and the ‘*R. ixodidis*’ strain investigated here, but ii) to be so with a counter-intuitively low degree of sequence identity of “>77%” [16], i.e. much lower than the pair-wise sequence identity values established across genus boundaries e.g. between specimens belonging to the *Rickettsiella* and the *Coxiella* or the *Legionella*. The two further sequences from Australian specimens were reported to be most homologous to the corresponding markers from the pathotypes ‘*R. armadillidii*’ (sample “Ita #162”, >92% sequence identity) and ‘*R. melolonthae*’ (sample “Ita #168”, >99% sequence identity). Similar values of, respectively, 75.4%, 93.6%, and 99.4% have in the present study been calculated for these pair-wise sequence identities from a p-distance matrix. However, phylogenetic reconstruction results presented here essentially coincide with those of the previous report [16] for sequences Ita #162 and Ita #168, but are contradictory with respect to sequence Ita #160: whereas the NJ phylogeny (not shown) places this *I. tasmani* derived sequence in close vicinity to the *R. grylli*, it clusters with sequence Ita #162 and ‘*R. armadillidii*’ sequences in the ML phylogeny (Figure 1). Moreover, the length of the respective branch seems to indicate a much faster evolution of the Ita #160 sequence as compared to the other 16 S rRNA sequences considered, and closer analysis of sequence alignments reveals a disproportionate homology distribution with extreme changes in block-wise sequence identity values along the Ita #160 sequence. One possible explanation of these findings would be that sequence Ita #160 might be a PCR artefact, possibly due to the fact reported by Vilcins et al. [16] that several ticks were pooled into sample Ita #160 prior to genetic analysis. Of course, a more detailed analysis of further sequence data would be needed to firmly determine the phylogenetic position of this *I. tasmani* derived bacterium. However, beyond these ambiguities and in view of the cladistic positions occupied within the *Rickettsiella* clade of Figure 1 by the *I. tasmani* derived strains Ita #162 and Ita #168, the presumed ‘*Diplorickettsia*’ strain from *I. ricinus* and the ‘*R. ixodidis*’ strain from *I. woodi*, it is getting sufficiently clear from our study that ixodid tick associated *Rickettsiella* bacteria do not form a tight phylogenetic cluster, but rather spread across the full range of the still ill defined diversity present within this gamma-proteobacterial genus.

In conclusion, 16 S ribosomal RNA based phylogenetic reconstruction complemented by likelihood based significance testing and multilocus sequence typing (MLST) leads to the firm taxonomic assignment of an intracellular bacterium isolated from a female culture of parthenogenetically multiplying hard ticks to the taxonomic genus *Rickettsiella*. Despite the currently existing inconsistencies in strain designation and species delineation within this genus, the *Ixodes* associated bacterium under study is further assigned to the taxonomic species *Rickettsiella grylli*. Moreover, the combined methodology employed, in particular the use of the *gidA* and *sucB* markers, is shown to provide potential for more informative taxonomic studies within the genus *Rickettsiella*. However, more respective sequence data are required to develop this potential into improved species and/or sub-species delineation within the genus.
